# Oral Health and Knowledge among Postpartum Women

**DOI:** 10.3390/children9101449

**Published:** 2022-09-22

**Authors:** Mordechai Ben David, Yaffa Callen, Hila Eliasi, Benjamin Peretz, Rasha Odeh-Natour, Michal Ben David Hadani, Sigalit Blumer

**Affiliations:** 1 Department of Obstetrics and Gynecology, Laniado Hospital, Netanya 42223, Israel; 2 Department of Paediatric Dentistry, The Maurice and Gabriela Goldshleger School of Dental Medicine, Faculty of Medicine, Tel Aviv University, Tel Aviv 69978, Israel; 3 Department of Nursing, Ruppin Academic Center, Emek Hefer 40294, Israel

**Keywords:** education, early childhood caries, neonates, oral examination, oral hygiene, pregnancy

## Abstract

Oral health behavior and risks during pregnancy and after birth affect the oral health of babies and toddlers. We examined the oral and gingival health and caries prevalence of 150 postpartum women shortly after giving birth and assessed their knowledge of oral hygiene using a questionnaire. We also compared the oral health knowledge of nulliparous and multiparous women. Although most participants (98.0%) understood the importance of maintaining oral hygiene in children, their overall knowledge of oral health was medium–low, regardless of the number of previous pregnancies. Only 4.6% of women received oral health advice from their obstetrician during their pregnancy. Most participants had a high gingival index score, which correlated with dental pain during pregnancy. In contrast, the number of decayed, missing and filled teeth was significantly lower in first-time mothers. There was a statistically significant positive correlation between women who regularly visit their dentist and those who regularly take their children to the dentist. Expecting mothers should be educated about their own oral health and that of their developing fetus and children. Raising awareness among obstetricians with regards to this topic may be an effective way to achieve this.

## 1. Introduction

Primary teeth start to develop as early as the second week of pregnancy and continue to develop and mineralize through birth [1]; therefore, gestational complications which affect the development of various organs may also affect tooth development. Maternal dental caries usually increases during pregnancy due to increased mouth acidity, a greater intake of sugary snacks (secondary to pregnancy cravings) and decreased attention to oral health maintenance [2]. Some women also have pregnancy gingivitis during their third trimester due to increased estrogen and progesterone levels [3], increasing the risk of developing periodontal disease, especially in those who were at risk prenatally. Maternal periodontal disease is a risk factor for premature births and low birth-weight babies [4,5], which in turn, harbor the risk of hypoplasia of the enamel [6,7], esthetic defects and caries. Peretz et al. reported that neonates born after a traumatic delivery or following maternal pregnancy complications, including vaginal bleeding, early contractions, infection or diabetes, were at a higher risk of developing early childhood caries (ECC) [8]. Therefore, maintaining oral health during pregnancy is essential [9].

Additional factors affect the oral health of babies and toddlers. During the first years of life, cariogenic micro-organisms, specifically *Streptococcus mutans* (SM), are passed down to the baby or toddler either vertically through the vagina of the mother or horizontally from family members and/or children in daycare [10,11]. Furthermore, there is a clear positive correlation between high levels of tooth decay in primary teeth and decay in permanent teeth in the same individuals [12]. Therefore, it is important to cultivate correct oral hygiene habits from a young age. This responsibility primarily falls on the parents of the child [13].

Guidelines developed by consensus meetings and expert panels following comprehensive reviews of the available evidence indicate that throughout pregnancy, it is safe to perform tooth extractions as well as preventive, diagnostic, restorative and periodontal procedures in order to maintain the oral health of mothers and their children [14]. According to these guidelines, dental examination should be performed every 6 months to maintain the oral health of pregnant women. Dental emergency treatments can be performed at any time during pregnancy, and dental diseases should be treated in a timely manner [14]. According to the American Dental Association (ADA) and the American College of Obstetricians and Gynecologists (ACOG), pregnant women should be counseled by both their obstetrician and dentist on the importance of good oral hygiene throughout pregnancy. Regular and emergency dental care, including the use of local anesthetics and radiographs, is safe at any stage during pregnancy when abdominal and thyroid shielding is used [2,15]. The authors suggest the avoidance of any treatment in the first trimester. Nevertheless, from an obstetric point of view, any examination (including X-rays) and any dental treatment can be performed during the entire pregnancy if necessary. According to the ADA and ACOG with regards to medications that can be taken during pregnancy, penicillin, amoxicillin, cephalosporins, clindamycin and metronidazole are safe for use in pregnant women as is local anesthesia, with or without epinephrine [2,16,17]. In general, the labeling of a drug should be consulted prior to its administration to an individual during pregnancy and the breastfeeding period. 

Many women do not pursue or receive any oral care during pregnancy, even if they experience signs or symptoms of oral disease [18,19,20,21], due to financial barriers and low awareness of the importance of oral health [18,21,22]. 

To understand the extent of knowledge that women have of oral health and best practices during pregnancy and as mothers to young children, we examined the oral and gingival health of women and their oral hygiene knowledge shortly after giving birth. We also compared oral health knowledge between primiparous and multiparous women. We hypothesized that multiparous women who have already given birth to several children would be more aware of issues regarding their own oral health and the oral health of the fetus/newborn and that they would take better care of their teeth and gums than primiparous women.

## 2. Materials and Methods

### 2.1. Study Design, Setting and Participants

We conducted a cross-sectional study among postpartum women, which was a collaboration between the Department of Pediatric Dentistry at Tel Aviv University and the Obstetrics and Gynecology Department of Laniado Hospital (Netanya, Israel). The study was approved by the institutional ethics committees of Laniado Hospital (protocol code 0120-17-LND, date of approval 30 January 2018) and Tel Aviv University (protocol code 34.18, date of approval 15 February 2018). 

Women who had given birth at Laniado hospital were approached up to 72 h after delivery and received an explanation about the study. All women who agreed to participate in the study signed an informed consent form.

### 2.2. Data Collection 

Data collection took place over 10 months and consisted of a questionnaire and a dental examination. During the rest hours of the hospital, the dentists checked women who had given birth in the prior 72 h. 

### 2.3. Questionnaire

Our priority was to administer a validated self-reported scale to measure the main variables in this study. However, as such a questionnaire was not found, we constructed a questionnaire based on our clinical experience and the literature. Although this questionnaire is not validated, it has a high face validity as it was examined by several colleagues.

Each woman completed a four-section questionnaire comprising 35 questions, most of which were multiple choice regarding: (1) participant demographics; (2) information about the pregnancy (high-risk, delivery type, birth weight); (3) the oral health of the participant (e.g., reason and date of last dental appointment, oral hygiene during the pregnancy, information on oral health provided by the obstetrician); (4) the knowledge and practices of the participant regarding pediatric oral hygiene. Overall, 11 of 35 questions evaluated the knowledge of the participant about oral health.

### 2.4. Oral Examination

After completing the questionnaire, each woman underwent a noninvasive oral examination performed by two independent dentists at her recovery bed using a flashlight, a mirror and a dental explorer. Three dental indices were indicated. The first index was the maternal decayed, missing and filled teeth (DMFT) [23], which is the sum of teeth that were affected by caries throughout the life of the participant. The plaque index (PI) [24] was determined by examining four different surfaces (mesial, distal, buccal and lingual) of six teeth (numbers 16, 12, 24, 44, 32 and 36). Each surface received a number between 0 (no plaque) and 3 (abundance of soft matter within the gingival pocket and/or on the tooth and gingival margin). The gingival index (GI) [25] also received a number between 0 (normal gum tissue) and 3 (severe inflammation/spontaneous bleeding of the gums). The total PI and GI were then summed up and divided by 6 (the number of teeth examined).

Following the oral examination, each participant was given an information sheet about maternal and pediatric oral health prepared by the affiliated Department of Pediatric Dentistry. 

### 2.5. Statistical Analysis

The collected data were entered and analyzed by SPSS statistical software version 15.0 (SPSS Inc., Chicago, IL, USA). Categorical variables were summarized by number and percentage, and continuous variables were summarized by mean and standard deviation (SD). The Shapiro–Wilk test was used to examine if the parameters were normally distributed. Those that were not normally distributed were divided into groups and turned into binary parameters according to the median of each parameter and compared using chi-squared test. A *p*-value of less than 0.05 was considered statistically significant. 

## 3. Results

A total of 150 women with a mean age of 30.24 ± 5.8 years (range, 19–43) were included in the study. Participant demographics, pregnancy and delivery characteristics are summarized in Table 1. Most participants had postsecondary education. A fifth of the women (20.7%) delivered their first child at the time of the study. A fifth of pregnancies (20.7%) were considered high risk. Most women (75.3%) had a natural vaginal delivery. The mean gestation week at birth was 39.4 ± 1.6, and the birth weight was 3325.6 ± 475.9 gr. Only three women (2.0%) delivered a premature baby. Most participants (88.7%) reported being under regular follow-ups during the current pregnancy.

Table 2 summarizes the answers of participants regarding their dental health and habits. Only a third of the participants (37.3%) reported visiting the dentist for a checkup every 6 or 12 months before their pregnancy, while the rest reported going only when experiencing oral pain. Most (78.0%) reported that they continued with their usual dental behavior during their pregnancy. Almost half of the participants (46.7%) had tooth or gum pain or both during the current pregnancy; however, 62% did not visit the dental clinic, even once, during their pregnancy, and 5.3% reported not going to the dentist because they believed that they could not undergo dental treatments while pregnant. Most patients (73.3%) did not visit a dental hygienist to have their teeth cleaned during pregnancy. 

Only 17.3% and 6.0% of the participants knew that their overall health and the health of their gums during pregnancy, respectively, can affect the teeth of their baby. Most participants (94.7%) did not receive oral health information from their obstetrician.

Most participants (98.0%) were aware of the importance of maintaining oral hygiene in children. About two-thirds (64.0%) answered that the toothbrush of a child should be replaced every 3 months, but 53.3% had no information about fluoride in the toothpaste of their child. When asked about the timing of the first dental examination, 3.3% answered that it should be performed when the child is 6 months old, 14.0% answered that it should be performed when the child is 12 months old, and 34.0% believed that this should be performed after all the teeth of the child had erupted. Over half of participants (53.3%) were not aware of sealants. Almost half of participants (48%) reported regularly taking their children for dental checkups, while 39.3% only take them if there is a problem, and 12% did not know if they should be taking them at all. About two-thirds of the participants (62.0%) obtain information about the dental care of children from the dentist and 22.7% from their friends, while the rest obtain such information from the media and the Internet. 

Most participants (74.7%) believe that children should only be given a limited number of sweet beverages and food. About two-thirds of participants (65.3%) put the eating spoon of their child in their mouths to sense its heat or taste the food before feeding the child, and over half (57.4%) put their child in bed for the night with a bottle, although 22.7% reported that the bottle only contains water. 

A higher percentage of primiparous participants received information from their obstetrician about the importance of maintaining oral health during pregnancy compared to multiparous participants (71.4% vs. 28.6%, *p* = 0.002). Contrarily, primiparous participants were less aware than multiparous children that they could visit the dental hygienist during their pregnancy (80% vs. 20%, *p* = 0.005). There were no statistically significant differences between these two groups regarding regular dental visits and the reason for the last dental visit.

The comparison of DMFT, PI and GI showed a statistically significant lower DMFT among primiparous women (*p* = 0.015) (Table 3), while PI and GI did not statistically differ. Participants who reported dental pain during their pregnancy had a significantly higher GI score (20 women, *p* = 0.038). No associations between GI scores and age, place of residence, education, the birth weight of newborns or the regular dental follow-ups of the participant were found.

Statistically significant positive correlations were found between participants who regularly visit the dentist and those who regularly take their children to the dentist (*p* < 0.0001) as well as those maintaining oral health during pregnancy (*p* = 0.016). In addition, women who regularly take their children to the dentist had significantly greater knowledge regarding the appropriate time for the first dental appointment for a child (*p* = 0.006). There was no significant difference between women who visited the dentist during pregnancy and the dental care they provided for their children (Table 4).

To evaluate the possible influence of various variables on the knowledge of the participants, we allotted a point for every correct knowledge question (N = 11). We then divided the women into three groups based on the number of points gained: high knowledge (HK): ≥8 points; medium knowledge (MK): 4–7 points; and low knowledge (LK): <4 points. Chi-squared tests were performed to examine the relationship between knowledge groups and demographic variables. 

**Figure 1:**
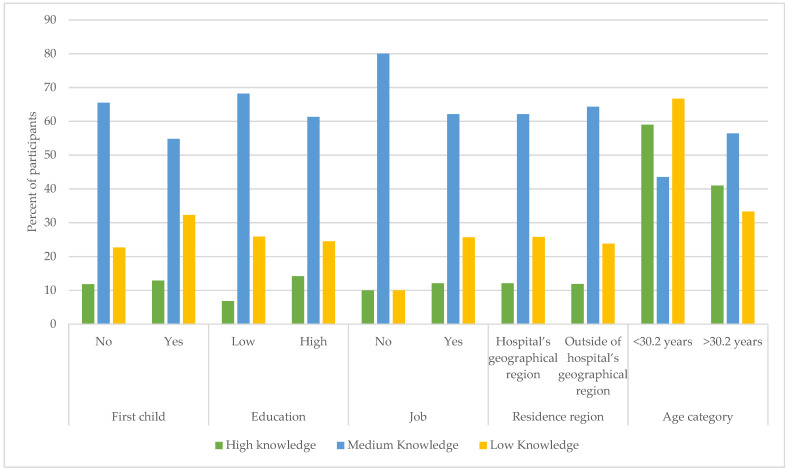
Relationship between demographic variables and knowledge of oral health among women.

The results show that overall, only 12% of participants correctly answered eight or more questions. As shown in Table 5 and Figure 1, only age was found to be statistically significantly associated with knowledge; women with more knowledge were significantly older than those with less knowledge (*p* = 0.014). The rest of the parameters were not statistically significantly different. 

To assess the overall effects of sociodemographic and pregnancy characteristics, oral health knowledge and habits on the outcome variables, logistic regressions were conducted. The results show that a late birth week is a risk factor for GI (OR = 1.40, [95% CI 1.06, 1.85). Poor oral health knowledge was also found to be a risk factor for GI (OR = 0.78, [95% CI 0.60, 1.07]) (Table 6).

Although no significant predictors were found for DMFT or PI, poor oral health habits and poor oral health knowledge were found to predict DMFT and PI (0.70 < OR < 0.89).

## 4. Discussion

Women are often unaware of the need to perform a general physical examination and a dental exam before becoming pregnant. In Israel, the birth book does not include a referral to a dentist or any other doctor unless there is an initial health problem. 

Although most participants (98.0%) understood the importance of maintaining oral hygiene in children, their overall knowledge of oral health during pregnancy and in young children was medium and low, respectively, regardless of the number of deliveries. Only the mean age was found to be statistically significantly associated with knowledge.

The pregnancy and postpartum periods are optimal times for health education as women are interested in information concerning the wellbeing of their newborns [26,27,28]. Healthcare professionals are the preferred source for health information among lay people regardless of their sociodemographic characteristics [29]. In the present study, most participants reported receiving information about oral health from their dentists. Despite the high percentage of women who regularly visited their obstetrician during their pregnancy, only 4.6% of them received information about oral health. Nulliparous women were more likely to receive such information. Although this may indicate a positive shift in the recognition of obstetricians of the importance of raising patient awareness regarding oral health and its impact on the fetus, oral health education should be extended to all pregnant women. 

In a survey conducted in 2008 in the United States (US) among 351 obstetricians, 84% of respondents recognized the importance of routine dental care during pregnancy, but 73% seldom asked pregnant patients whether they had recently seen a dentist, 54% did not ask about the current oral health of the patient, 69% did not offer information about oral care, and 38% did not advise their pregnant patients to see a dentist for routine preventive care [30]. Similarly, in a study conducted among French obstetricians, most were aware of the inflammatory nature of periodontal diseases and its adverse impact on pregnancy outcomes, but only 26% asked their patients about oral health, only 10.5% systematically provided information on oral health, and only 33% systematically referred the patient to a dentist [31]. To ensure that health professionals and pregnant women are aware of the importance of receiving oral health care during pregnancy, US federal agencies and organizations have initiated programs, promoted policies and provided education and training [32]. Furthermore, in British Colombia, Canada, it has been suggested to integrate preventive oral health into routine prenatal care [21].

Dental examinations performed among the study population up to 72 h after birth have shown that most study participants had a high GI score. Only dental pain during pregnancy was positively correlated with GI, suggesting that the pain these women experienced during pregnancy is most likely related to early reversible disease (gingivitis) or degenerative disease (periodontitis) during pregnancy. This finding is consistent with elevated hormone levels during pregnancy that make the blood vessels more permeable, causing edema and inflammation [3]. Although this finding is consistent with what we expected to find, it is nonetheless alarming due to its ability to affect the teeth of the developing fetus, and the finding that most participants were unaware of it. A survey conducted among pregnant women in the US showed that 65% of pregnant women did not visit their dentist at all during their pregnancy, and less than half of pregnant women with oral pain visited their dentist. The most common reason for avoiding visiting the dentist was fear of harming the fetus [28]. The treatment of periodontal diseases in pregnancy, which is commonly performed by scaling and root planning to remove calculus, is considered safe [33,34,35,36] and usually improves maternal oral health [35,36]. 

Although the results of the clinical dental exam were similar among all participants, DMFT was significantly lower in first-time mothers. This may be attributed to their overall younger age or to their lower number of pregnancies. As previously stated, there is an increase in dental caries during pregnancy due to the increased acidity from vomiting and increased sugar intake [2].

We found that there were statistically significant positive correlations between women who regularly take their children to the dentist and women who regularly visit their dentist as well as those who maintained oral health during pregnancy. Parents with high oral hygiene awareness take better care of the teeth of their children and, on average, had more knowledge regarding the appropriate time for the first dental appointment of a child. Baker et al. found that beliefs about infant oral health care were influenced by maternal oral health beliefs and access to dental care but were not related to birth history. The topic of early habits is significant as children with dental caries are at a higher risk of developing caries in their permanent teeth [12]. 

The collective knowledge of the participants was alarmingly low. Only 12% correctly answered eight or more questions. This finding is in line with studies performed in other countries [37,38,39]. Wapniarska et al. found that knowledge in parents regarding oral health was very low, especially in young parents [38]. A survey conducted among 94 mothers at a medical center in New York revealed that most had no knowledge about the vertical transmission of bacteria such as SM, and many were unaware that the AAPD recommends that the first dental exam of a child should be performed before 12 months of age [40]. In a study conducted in the city of Durg, Chhattisgarh (India), knowledge of and attitudes about the effect of the oral health of expecting mothers and its impact on infant oral health was poor and significantly varied by age, trimester, the number of pregnancies and education level [37]. Similarly, a study in a maternity hospital in Kuwait among 430 pregnant women also revealed poor knowledge of ECC. Age, the number of children, education, the frequency of dental visits and flossing frequency were significantly related to ECC knowledge levels. 

Our study revealed that older age was positively correlated with oral health knowledge. Al-Sane et al. also found that expecting mothers ≥35 years had significantly greater knowledge about ECC [41]. This is plausible as older women most probably have older children; therefore, their previous experience may affect their knowledge. Baker et al. found that multiparous women are more knowledgeable about the oral health of infants compared to nulliparous or primiparous women, but the maternal beliefs about oral health of infants were comparable among the groups [42]. Nevertheless, we, and others, did not find a relationship between oral health knowledge and the number of deliveries. In addition, other studies have not found a relationship between the oral health knowledge of expectant mothers and age [42,43,44]. 

We did not find a correlation between education and oral health knowledge. Przeklasa-Bierowiec et al. reported that pregnant women with higher education levels were better informed regarding the prevention of oral diseases, but their knowledge was still not satisfactory, and most women lacked awareness of oral disease prevention [45]. 

A review of the knowledge questions revealed knowledge gaps indicating the areas in which pregnant women should be educated. Less than 18% correctly answered the question regarding the timing of the first dental visit. Additionally, most women believed that demonstrating how to brush teeth is sufficient for the dental care of a child, while, in fact, the motor skills required to adequately brush teeth are only developed at around 7 years of age. Most women understood the harmful effects of sweet foods but were unaware of the detrimental affect the time of day of the consumption has on the teeth of children. Over half of women said that the food of a child should be tasted before feeding to ensure the correct food temperature. When asked about putting their child to bed with a bottle, 42.4% answered that they do not do this due to the harmful effects on the teeth of their child. Over 50% of mothers did not have knowledge regarding fluoride in toothpaste and sealants for children which are important factors in decreasing and preventing caries, which highlights the need to educate parents of their effects. 

The limitations of the study include its cross-sectional design which only enabled us to evaluate the clinical and behavioral parameters at a single time-point and by its convenience sampling method, which may have impacted the generalizability of the findings. The self-reporting nature of the study may have led to a social desirability bias when completing questions on maintaining oral health which may have led to the over-reporting of maintaining oral health or going to dental checkups. It is also important to note that the questionnaire is not validated; however, it has a high face validity as it was examined by several colleagues. Prior to performing the oral examinations, the dentists reviewed together the exams that were to be undertaken, but inter-examiner reliability was not calculated. In addition, the study was performed in a single hospital, although the diversity of the women who were tested was unaffected as only 43.7% of participants were from the city in which the hospital is located. We did not evaluate the socioeconomic status of the women, which was previously reported to be negatively associated with oral health and dental disease [46,47,48,49]. The uniqueness of this study is that it examined both the oral health knowledge and the behavior of the participants in parallel to a clinical oral exam.

## 5. Conclusions

Our study stresses the importance of educating expecting mothers about the oral health of their developing fetus and their children as well as their own oral health. Governmental programs should be established to raise awareness of this topic and educate young people, especially women of child-bearing age, about good oral health practices and the importance of having physical and oral examinations when planning a pregnancy. International professional association guidelines such as those of the ADA and ACOG should be followed. As most pregnant women have regular pregnancy follow-ups, awareness of the importance of oral health should be raised among gynecologists and obstetricians, and they should be encouraged to provide this information to their patients as early as possible, even when discussing family planning. This information should also be made available at prenatal clinics and mother-and-child health clinics. An oral health specialist should be part of the prenatal clinic team. Alternatively, nurses at such clinics should undergo training in oral health education and actively ask the women about their oral health and explain the importance of regularly seeing a dentist. Pregnant women should be aware that if they experience oral pain, bleeding or inflammation, they should see a dentist. Finally, dentists should also speak to women about the importance of regular dental checkups and their safety during pregnancy. 

Improving the oral health knowledge of expecting mothers may improve maternal and pediatric oral hygiene and reduce future healthcare costs. 

## Figures and Tables

**Table 1:** Participant demographics, pregnancy and delivery characteristics.

	Study Population N = 150
**Age, years**	30.24 ± 5.8
**Education**	
Up to upper secondary education	44 (29.3%)
Postsecondary nontertiary or tertiary education, bachelor’s degree or higher degree	106 (70.7%)
**Birth number**	
1	31 (20.7%)
2	40 (26.7%)
3	30 (20.0%)
≥4	49 (32.7%)
**High-risk pregnancy**	31 (20.7%)
**Delivery**	
Vaginal/natural	118 (75.3%)
Caesarian section	25 (16.7%)
Instrumental	10 (6.7%)
**Gestation week at birth**	39.4 ± 1.6
**Birth weight, gr**	3325.6 ± 475.9
**Premature birth**	3 (2.0%)
**Regular follow-ups during pregnancy**	133 (88.7%)

Categorical variables are shown as numbers (percent), and continuous variables are shown as mean ± standard deviations.

**Table 2:** Oral health behaviors and knowledge of best oral practices of participants.

Question	Study Population N = 150
**Oral Health Behaviors**	
**Do you visit the dentist regularly for check-ups?**	
Every 6 months	18 (12.0%)
Every 12 months	38 (25.3%)
Only when in pain	93 (62.0%)
**Did you visit the dentist during the current pregnancy**	
Once	36 (24.0%)
Regularly	12 (8.0%)
No	93 (62.0%)
I did not go to the dentist because I thought one cannot undergo dental treatments while pregnant	8 (5.3%)
**Did you have a toothache or suffer from gum pain during the current pregnancy?**	
Toothache	28 (18.8%)
Gum pain	21 (14.1%)
Both toothache and gum pain	21 (14.1%)
I did not have pain during the pregnancy	79 (53.0%)
**Did you undergo tooth cleaning by a dental hygienist or a dentist during the current pregnancy?**	
Yes	34 (22.7%)
No	110 (73.3%)
I did not know you could visit the dental hygienist while pregnant	5 (3.3%)
**Did you maintain the health or your teeth and gums during the current pregnancy**	
Yes, more than usual	22 (14.7%)
No; it was difficult to do so because of all the other pregnancy-related examinations	10 (6.7%)
My dental behavior was as usual	117 (78.0%)
**Did your obstetrician-gynecologist give you an explanation about the importance of maintaining oral health (teeth and gums) during pregnancy**	
Yes	7 (4.7%)
No, the obstetrician-gynecologist did not mention this issue	142 (94.7%)
**Do you taste the food in your child’s feeding spoon before feeding him/her? (or intend to taste—if it is your first child)**	
Yes, it is necessary for sensing the food’s temperature	90 (60.0%)
Yes, to see if the food is tasty	8 (5.3%)
Never	51 (34.0%)
**Did your older children have their teeth sealed (or do you intend your children to undergo this procedure)?**	
Yes, it is important	32 (21.3%)
No	36 (24.0%)
I don’t know this procedure	80 (53.3%)
It doesn’t matter to me	2 (1.3%)
**From whom do you obtain information about your child’s dental care?**	
The media: newspapers, television	11 (7.3%)
The internet	11 (7.3%)
The dentist	993 (62.0%)
Friends	34 (22.7%)
**Knowledge on Oral Health Best Practices**	
**Does your health during pregnancy affect your baby’s teeth**	
Correct response	26 (17.3%)
Incorrect response	123 (82.7%)
**Does your health during pregnancy affect your baby’s teeth**	
Correct response	9 (6.0%)
Incorrect response	140 (94.0%)
**When should you brush your child’s teeth**	
Correct response	138 (92.0%)
Incorrect response	11 (8.0%)
**When should you replace your child’s toothbrush?**	
Correct response	96 (64.0%)
Incorrect response	53 (36.0%)
**Is it important to maintain the oral hygiene of children?**	
Correct response	60 (40.0%)
Incorrect response	88 (60.0%)
**At what age should children undergo their first dental examination?**	
Correct response	5 (3.3%)
Incorrect response	145 (96.7%)
**When is the best time to give your child sweet beverages or food?**	
Correct response	18 (12.0%)
Incorrect response	131 (88.0%)
**Do you put your child to bed at night with a bottle?**	
Correct response	34 (22.7%)
Incorrect response	116 (77.3%)
**Do you have information on fluoride in your child’s toothpaste?**	
Correct response	47 (31.3%)
Incorrect response	102 (68.7%)

**Table 3:** Oral examination results by number of deliveries.

		Delivery			
		1 N = 31	>1 N = 87	Total N = 150	*p*-Value
DMFT	Low	29 (25.0%)	87 (75.0%)	116 (100%)	0.015
	High	2 (5.9%)	32 (94.1%)	34 (100%)	
GI	Low	3 (12%)	22 (88%)	25 (100%)	0.255
	Medium	2 (11.8%)	15 (88.2%)	17 (100%)	
	High	26 (24.1%)	82 (75.9%)	108 (100%)	
PI	Low	7 (21.2%)	26 (78.8%)	33 (100%)	0.339
	Medium	3 (10.7%)	25 (89.3%)	28 (100%)	
	High	21 (23.6%)	68 (76.4%)	89 (100%)	

DMFT, decayed, missing and filled teeth; GI, gingival index; PI, plaque index.

**Table 4:** Differences between women who regularly take their children to dental checkups and those who do not regularly take their children to dental checkups.

		Takes the Children to Regular Dental Checkups	Does Not Take the Children to Regular Dental Checkups	Does Not Know	Total	*p*-Value
Visits the dentist regularly for check-up	Yes	17 (94.4%)	1 (5.6%)	0 (0.0%)	18 (100%)	0.000
	Sometimes	23 (60.5%)	10 (26.3%)	5 (13.2%)	38 (100%)	
	No	33 (35.1%)	48 (51.1%)	13 (13.8%)	94 (100%)	
Visited the dentist during pregnancy	Yes	28 (58.3%)	17 (35.4%)	3 (6.3%)	48 (100%)	0.448
	No	41 (43.6%)	39 (41.5%)	14 (14.9%)	94 (100%)	
	Thought it was not allowed	4 (50.0%)	3 (37.5%)	1 (12.5%)	8 (100%)	
Maintains her oral health during pregnancy	More than usual	18 (81.8%)	4 (18.2%)	0 (0.0%)	22 (100%)	0.016
	No	4 (40.0%)	4 (40.0%)	2 (20.0%)	10 (100%)	
	As usual	51 (43.2%)	51 (43.2%)	16 (13.6%)	118 (100%)	
When should you take your child for his/her first dental check-up	Correct	20 (76.9%)	4 (15.4%)	2 (7.7%)	26 (100%)	0.006
	Incorrect	53 (42.7%)	55 (44.4%)	16 (12.9%)	124 (100%)	

**Table 5:** Oral health knowledge by number of births, education, job, residence region and age.

		Knowledge n (%)				
		HK	MK	LK	Total	*p*-Value *
	Total	18 (12.0%)	95 (63.3%)	37 (24.7%)	150 (100%)	
First child	No	14 (11.8%)	78 (65.5%)	27 (22.7%)	119 (100%)	0.500
	Yes	4 (12.9%)	17 (54.8%)	10 (32.3%)	31 (100%)	
Education	Low	3 (6.8%)	30 (68.2%)	11 (25.0%)	44 (100%)	0.443
	High	15 (14.2%)	65 (61.3%)	26 (24.5%)	106 (100%)	
Employed	No	1 (10.0%)	8 (80.0%)	1 (10.0%)	10 (100%)	0.487
	Yes	17 (12.1%)	87 (62.1%)	36 (25.7%)	140 (100%)	
Residence region	Geographical region of hospital	8 (12.1%)	41 (62.1%)	17 (25.8%)	66 (100%)	0.958
	Outside of geographical region of hospital	10 (11.9%)	54 (64.3%)	20 (23.8%)	84 (100%)	
Age above or below the mean age of the study population (30.2 years)	<30.2 years	46 (59.0%)	30 (43.5%)	2 (66.7%)	78 (100%)	0.014
	>30.2 years	32 (41.0%)	39 (56.5%)	1 (33.3%)	72 (100%)	

* *p*-value by chi-squared test.

**Table 6:** Odds ratio predicting DMFT, PI and GI according to sociodemographic, pregnancy, oral health habits and knowledge.

Variable	DMFT		PI		GI	
	OR	95% CI	OR	95% CI	OR	95% CI
Age	1.05	(0.98–1.14)	0.95	(0.88–1.03)	0.99	(0.92–1.07)
Education	0.91	(0.41–1.98)	1.79	(0.82–3.93)	1.51	(0.68–3.37)
Child number	1.07	(0.88–1.30)	1.08	(0.91–1.30)	1.06	(0.89–1.28)
Birth week	1.00	(0.78–1.28)	1.08	(0.84–1.39)	1.40 *	(1.06–1.85)
Birth weight	1.00	(1.00–1.00)	1.00	(1.00–1.00)	1.00	(1.00–1.00)
Oral health habits	0.82	(0.50–1.35)	0.76	(0.45–1.12)	0.70	(0.43–1.16)
Oral health knowledge	0.86	(0.76–1.22)	0.89	(0.70–1.13)	0.78 *	(0.60–1.07)

CI, confidence interval; DMFT, decayed, missing and filled teeth; GI, gingival index; OR, odds ratio; PI, plaque index. * *p* < 0.05.

## Data Availability

Data are available upon reasonable request from the corresponding author.
